# Expanding Understanding of Autism Spectrum Disorder in Girls and Women: A New Paradigm

**DOI:** 10.3390/bs16030396

**Published:** 2026-03-09

**Authors:** Dylan Rose Bitensky, Caroline S. Clauss-Ehlers, Kyle M. Veksler

**Affiliations:** Department of Psychology, School of Health Professions, Long Island University Brooklyn, Brooklyn, NY 11201, USA; dylan.bitensky@my.liu.edu (D.R.B.); kyle.veksler@my.liu.edu (K.M.V.)

**Keywords:** autism spectrum disorder, sex-differences, neurodiversity paradigm, female autism phenotype, Multimodal Autism Assessment for Girls and Women (MAAGW)

## Abstract

The definition of Autism Spectrum Disorder (ASD) has evolved since the diagnosis was first conceptualized. However, past and current understandings of ASD have largely been shaped by research focused on individuals assigned male at birth. This has influenced current diagnostic criteria, which often fail to capture female presentations and likely contribute to lower reported prevalence rates in girls. Recognizing female-specific presentations is critical to reducing misdiagnosis and underdiagnosis; therefore, this paper reviews research on how the DSM-5-TR two-factor model applies to girls. We then describe how female presentations of ASD are characterized by distinct cognitive profiles and frequent use of camouflaging to mask typical ASD symptoms, particularly social difficulties, which can obscure clinical presentation and delay diagnosis. Subsequently, we evaluate how current assessments and accommodations may fail to address the needs of females. Clauss-Ehlers introduces the Multimodal Autism Assessment for Girls and Women, which emphasizes the importance of assessing girls within an appropriate contextual lens. Finally, the paper calls for a gender-focused neurodiversity paradigm that highlights differences in presentation, promotes an approach focused on strength-based interventions, and outlines directions for future research and clinical implications based on this framework.

## 1. Historical Considerations in Defining Autism

To begin, it is important to note that there have been enormous changes in our understanding of Autism Spectrum Disorder (ASD), particularly since ASD was introduced in the 2013 Diagnostic and Statistical Manual of Mental Disorders, 5th edition (DSM, American Psychiatric Association, 2013). Previously, what we now call ASD was presented under different diagnoses such as autistic disorder, Asperger syndrome, high functioning autism, atypical autism, and pervasive developmental disorder (Parritz & Troy, 2024).

Autism was reported in the literature as early as the 1940s, when Kanner (1943) published a paper titled “Autistic Disturbances of Affective Contact”, which presented cases of children who engaged in extreme isolating behaviors. However, reports were also made in the 1700s and 1800s (Rosen et al., 2021). Kanner’s (1943) early description was important in helping people differentiate autism from schizophrenia, which had been a differential diagnostic issue. Despite Kanner’s (1943) diagnostic differential, as recently as 1968, “childhood schizophrenia” was the only DSM-II category related to developmental considerations described by Kanner (1943; American Psychiatric Association, 1968). It was not until 1980 that the DSM-III (American Psychiatric Association, 1980) included autism as a diagnostic category, which looked at autism as a developmental disorder, specifically, a Pervasive Developmental Disorder (PDD).

While a review of the history of autism is beyond the scope of this article, the paragraphs that follow will show how a male-centered history in autism assessment contributes to the underdiagnosis of autism among females today. For instance, Kanner’s (1943) early assessment of autism was based on observations of characteristics that may be more prevalent in boys (e.g., rigid routines, repetitive movement) rather than focusing on how girls and women might mask social behaviors. In fact, the observations Kanner documented in his 1943 “preliminary report” (p. 217) were based on a “presentation of the case material” (p. 217) of 11 children, 9 of whom were boys and only 2 of whom were girls.

Interestingly and unbeknownst to Kanner (1943), the case presentations of the two girls, Barbara K. (Case 5) and Elaine C. (Case 11), foreshadow how girls and women may present differently than males, leading to their underdiagnosis within a historically male-centric context. For instance, Barbara K.’s father describes her behaviors in ways that we now understand as masking and camouflage, evidenced by mimicking social behaviors to fit in. Barbara K.’s father says: “In camp last summer she was well liked, learned to swim, is graceful in water (had always appeared awkward in her motility before), overcame fear of ponies, played best with children of 5 years of age. At camp she slid into avitaminosis and malnutrition but offered almost no verbal complaints” (Kanner, 1943, p. 229).

For Elaine C., after being put in a private school due to her symptoms, her father reported “rather amazing changes” when he says that Elaine’s “clear eyes that have long since lost any trace of that animal wildness they periodically showed in the time you knew her. She speaks well on almost any subject, though with something of an odd intonation. Her conversation is still rambling talk, frequently with an amusing point, and it is only occasional, deliberate, and announced” (Kanner, 1943, p. 241).

This history and the potential for misconceptions inform the purpose of the current article, which is to help clarify how ASD presents in girls and women by deepening our understanding of how criteria manifest in girls and women, and to underscore that accurate diagnosis is essential for access to appropriate and effective intervention. If girls and women with ASD are misdiagnosed or overlooked, the treatment they receive may focus on secondary symptoms rather than on the underlying neurodevelopmental profile that shapes symptomatology. For example, interventions may target anxiety-related symptoms, which can be beneficial to an extent, but fail to address core features such as subtle social challenges, including difficulty interpreting implicit social cues, that may be obscured by masking and an outward appearance of social competence. Further, a misdiagnosis among girls and women may not only contribute to ineffective treatment but can also lead to years of treatment for other mental health issues that do not reflect or correspond with the individual’s concerns (McQuaid et al., 2024; Tamilson et al., 2025). The girl or woman with undiagnosed ASD, who is instead misdiagnosed with an issue such as Borderline Personality Disorder, Anxiety Disorders, and other diagnoses, may very likely feel stigmatized and invalidated for an experience that has yet to be adequately named (McQuaid et al., 2024; Tamilson et al., 2025). The potential for such deleterious outcomes speaks to the importance of accurate identification, diagnosis, and subsequent intervention for girls and women with ASD. The following paragraphs thus provide a contemporary diagnosis of ASD, followed by the presentation of a neurodiversity framework. Through a consideration of the underdiagnosis of girls and women, this framework will be applied as a neurodiversity paradigm that seeks to be responsive to the presentation of ASD in girls and women and how a more nuanced understanding of ASD in girls and women can inform treatment approaches described below.

## 2. Methodology

The conceptual framework for the current article was to explore how a male-centered history of understanding, diagnosing, and treating ASD contributes to girls and women being misunderstood, underdiagnosed, and not receiving treatment as a result. A narrative review process is the methodological approach employed, given its focus on summarizing findings across various topics. This is in contrast to a systematic review, which often focuses on one particular question (Sukhera, 2022). Given that the current review addressed multiple considerations, with a focus on underdiagnosis, misdiagnosis, and resulting treatment, a narrative frame, which encompasses a broader range of considerations, was deemed more applicable than a systematic review (Sukhera, 2022).

Further, although systematic reviews often employ the 2020 Preferred Reporting Items for Systematic Reviews and Meta-Analyses (PRISMA) framework, the current narrative review consulted aspects of the PRISMA checklist, specifically applying several items from the Methods section and topic to our narrative approach (Sukhera, 2022; Page et al., 2021). For instance, in response to PRISMA 2020 expanded checklist item 5, “specify the inclusion and exclusion criteria for the review and how studies were grouped for synthesis,” the authors specifically focused on sex-based differences in presentation, diagnosis, assessment practices, and accommodations for those with ASD. Additionally, authors sought out studies focusing on girls and women with ASD who show average to above-average cognitive and language abilities, but whose presentations are often overlooked due to masking, camouflaging, and less overt symptom expression (Page et al., 2021). In accordance with checklist item 6, which asks to identify “databases, registers, websites, organizations, reference lists, and other sources,” authors searched for relevant studies and information primarily using the PubMed database and Google Scholar. In addition, Henderson’s table was included with permission (Henderson, 2026). Regarding checklist item 7’s focus on search strategy, the search terms included “autism” OR “autism spectrum disorder” OR “ASD” (AND female OR women OR girls) AND (differences OR variability OR phenotype OR characteristics). Additional searches included related terms such as camouflaging, masking, sex differences, gender differences, assessment, diagnosis, restricted and repetitive behaviors, cognitive profiles, misdiagnosis, and accommodations to ensure coverage of key domains addressed in the current article (Page et al., 2021; Sukhera, 2022).

To eliminate potential biases in the screening for papers to be included/excluded in this narrative review, all three authors were involved in the screening process. The inclusion criteria included peer-reviewed studies, clinical sample data, meta-analyses, seminal theoretical papers, diagnostic criteria, and information relevant to today’s clinical practices and understanding of girls and women with ASD, such as sex-based differences in presentation, diagnosis, assessment practices, and accommodations for those with ASD. Papers were excluded if they did not meet the criteria above, fit the scope of the paper, or if the information was inapplicable to the research topic. Initial screenings for relevance were based on title and abstract, after which the full-text versions were retrieved, potentially being excluded if they did not fit the criteria. Citation tracking was utilized by reviewing the reference lists of articles that met the inclusion criteria. All articles were organized thematically rather than chronologically to fit the narrative review style.

## 3. Defining Autism Spectrum Disorder

Autism spectrum disorder (ASD) is characterized by difficulties with social reciprocity, social communication, flexibility, and sensory processing (American Psychiatric Association, 2013). Many children with ASD also experience motor difficulties, such as unusual gait, slower pace, decreased step length, increased knee flexion, and unusual upper-extremity positions during walking (Damasio & Maurer, 1978; Vilensky et al., 1981). According to the Diagnostic and Statistical Manual of Mental Disorders (DSM-5-TR; American Psychiatric Association, 2022, 5th ed., text rev.), a person must demonstrate persistent deficits in each of the three areas of social communication and interaction and a minimum of two of the four types of restrictive, repetitive behaviors (RRBIs). Alternatively, for individuals with ASD, there are three levels of severity for each criterion, with levels of severity indicating the amount of support needed. An individual on the spectrum with Level 1 requires the least support, while Level 3 requires the highest level of support. Supports include basic life skills, academic activities, various prompts, and independence skills. Appendix A presents a visual depiction of the criteria along with a description of the severity levels.

As shown in Appendix A, the current ASD diagnostic criteria indicate that an individual must demonstrate all three persistent deficits in social communication and social interaction and at least two of four restrictive, repetitive behavior patterns, interests, or activities (RRBIs); and that these must be present in the early developmental years (but may not become apparent until there are significant social demands on someone), that the symptoms have a significant negative impact on the individual’s functioning, and that what is being seen is not due to a developmental delay or an intellectual developmental disability. Further, the criteria state that if autism is comorbid with an intellectual developmental disability, which may occur, social communication needs to be below expectations for the developmental period. While some research has shown that this two-factor criterion for diagnosing autism has led to a more focused way to diagnose autism in children (Wiggins et al., 2019), there continue to be questions that emerge.

For instance, Criterion C states that symptoms must be present in the early developmental years but may not become apparent until the later years. Perhaps social interactions are manageable for an individual in the early years, with scaffolding from teachers and parents, but become increasingly problematic in adolescence and young adulthood, as adults step back from their scaffolding roles as children become young adults. In the context of Criterion C, alongside the later emergence of social difficulties, what might actually be problems with social communication and interaction related to someone with undiagnosed ASD may mistakenly be labeled as issues related to adolescent development, Borderline Personality Disorder, or Major Depressive Disorder (McQuaid et al., 2024; Tamilson et al., 2025). The question here is developmental: If symptoms do not appear until later, how can we determine if ASD is a concern during the early years? Furthermore, if Criterion A symptoms begin to emerge in social settings where the young adult is expected to be more independent in social interactions, how can we then understand that these might be symptoms of ASD and not another disorder or developmental issue?

In another example, the criteria discuss how ASD and intellectual disability may frequently co-occur. While the focus on comorbidity is important, it does not necessarily consider the young person who is twice-exceptional, meaning the young person has ASD and is also intellectually gifted. Here, the child may not be diagnosed with ASD if their intellectual abilities camouflage other criteria that present for ASD. For instance, if a student presents as highly intelligent but struggles with making friendships and is consistently isolated over time, this dynamic might be misinterpreted as the young person being “book smart” and just not social, rather than having ASD. These complexities may result in the reality that some children may go undiagnosed. The following paragraphs highlight how this appears to be the case for females, with girls, young women, and women being diagnosed less than boys, young men, and men (Schuck et al., 2019; Ochoa-Lubinoff et al., 2023).

## 4. Demographic Differences in the Diagnosis of ASD Between Girls and Boys

The Autism and Developmental Disabilities Monitoring Network is an organization that works to assess the prevalence rates of ASD and related characteristics among 4- to 8-year-olds (Shaw et al., 2025). In 2022, there were 16 sites that helped determine ASD prevalence, located in Arizona, Arkansas, California, Georgia, Indiana, Maryland, Minnesota, Missouri, New Jersey, Pennsylvania, Puerto Rico, Tennessee, two sites in Texas (e.g., Austin and Laredo), Utah, and Wisconsin. These researchers found that the prevalence rate was consistently higher for boys than for girls across sites, with a significant difference in the number of boys diagnosed with ASD in comparison to the number of girls. For instance, the study found that for every 1000 children in the study, 49.2 boys were diagnosed with ASD in comparison to 14.3 girls. This makes the rate of ASD 3.4 times higher in boys than in girls (Shaw et al., 2025). Further, a similar ratio of prevalence rates in ASD was found when meta-analyses include individuals ages 0–18, displaying a 3:1 male to female ratio in diagnosis (Loomes et al., 2017).

It is estimated, however, that the true ratio is probably smaller due to the knowledge that many girls go undiagnosed. For instance, this particular meta-analysis looked at both active studies, meaning those that screened the entire population for ASD traits regardless of an existing diagnosis, and passive studies, those that only included those that already had a formal ASD diagnosis. In active studies, there were 24 girls for every 100 participants identified with ASD traits, whereas in passive studies, there were only 18 girls for every 100 participants (Loomes et al., 2017). The higher proportion of girls with ASD seen in active studies demonstrates how girls in the community may display ASD traits but never go diagnosed. Moreover, assessments used in both active and passive studies may not be capturing female presentations of ASD to their full extent. Still, prevalence differences between men and women speak to the need for a more complex understanding of how ASD presents for girls and women in comparison to boys and men to prevent girls from going misdiagnosed/undiagnosed and delaying treatment. Thus, the following paragraphs present gender considerations related to differences in social and cognitive symptomatology and implications for our greater understanding of ASD.

## 5. Gender Considerations Related to the Two-Factor Model as Well as Social and Cognitive Symptomatology: Implications for ASD

Historically, most research on ASD has focused on males, shaping our understanding of the disorder around how it typically presents in boys and men (de Giambattista et al., 2021). For instance, D’Mello et al. (2022) found that research studies on ASD either enroll small numbers of women as participants or simply do not include women in ASD research. They talk about how research uses inclusion/exclusion criteria to confirm that someone has ASD before being enrolled in the study. Their research, however, has found that researchers who have used the Autism Diagnostic Observation Scale (ADOS; Lord et al., 2000) as a way to determine if someone has autism so as to be included in the study led to girls and women with ASD being 2.5 times more likely to be excluded for study participation in comparison to men with ASD (D’Mello et al., 2022). As a result, symptoms more common in girls and women have often been overlooked or misunderstood (D’Mello et al., 2022). However, recent studies have begun to highlight significant differences in how ASD manifests across sexes (Schuck et al., 2019). These differences carry important implications for diagnosis and suggest that interventions should be tailored to better address ASD’s diverse presentations.

Overview and application to DSM-5-TR criteria. Recent literature has focused on how the two-factor model of ASD that encompasses social and communication difficulties and repetitive behaviors/fixed interests may manifest in nuanced ways for girls and women. For instance, Dr. Donna Henderson notes that what people typically think about when considering ASD criteria may actually reflect an entirely different significance (Henderson, 2026; Henderson et al., 2023). Furthermore, when the criteria are applied to girls and women in ways we might not consider, problems related to the two-factor model may emerge in very specific ways.

For instance, Henderson et al. (2023) share how, for nonverbal communication problems, people tend to think about someone with ASD as having no eye contact. These authors go on to say, however, that the real meaning of this nonverbal communication for people with ASD is that eye contact is used in attempts to manage social interactions (Henderson et al., 2023). Hence, as indicated in Table 1, it’s important to ask specifically about what eye contact means. Questions that one might ask in this area include: Is it easy to engage in eye contact but does not feel comfortable? Is it part of camouflaging in an attempt to fit in? Does it feel forced even if it can be sustained? This is but one example of how an ASD criterion may play out and in contrast to our own assumptions of what a behavior might represent. Table 1 demonstrates how other criteria within the ASD two-factor model might present (Henderson, 2026). 

Social skills and autism. A key difference in presentation across sexes lies in socio-communicative presentation. Girls and women with ASD often show subtler social and communication difficulties, frequently through masking or compensation, which contributes to under- and misdiagnosis (Paolizzi et al., 2025). The camouflage hypothesis helps explain this pattern: to meet social expectations, many girls hide autistic traits, shaping peer relations and bullying risk, impacting the time of diagnosis (Cook et al., 2018).

This hypothesis further gives evidence to the female autism phenotype that describes female-specific traits of autism, which differ from the main male-based conceptualization of ASD (Bargiela et al., 2016). One example of the female autism phenotype is higher social motivation among girls and women with ASD than boys; many report wanting friends and to “fit in,” which often leads to masking behaviors (Sedgewick et al., 2016). These masking behaviors contribute to many girls and women with ASD ‘camouflaging’ some of their autistic traits through imitating peers’ mannerisms or voices, consciously adjusting their personas, or choosing not to disclose a diagnosis to avoid stigma (Cook et al., 2018). Camouflaging also clarifies why studies sometimes find fewer obvious social-behavioral differences among girls and women with ASD: many remain physically close to peers and engage in brief, fleeting interactions that appear typical on the surface but are non-neurotypical in content (Øien et al., 2017; Dean et al., 2017).

Studies describe girls and women who ‘pretend to be normal’ or ‘camouflage’ by developing neurotypical-seeming social presentations through carefully observing peers, reading novels and psychology books, imitating fictional characters, and learning through trial and error in social situations (Bargiela et al., 2016). However, this masking or camouflaging can obscure support needs, contributing to delayed or missed diagnosis, internalized distress (e.g., somatic symptoms), and elevated anxiety. This masking of autistic traits often leads parents to hear from schools that their daughter is ‘fine’ despite significant difficulties at home (Cook et al., 2018), impairing any intervention that may be given to someone who presents in a more classical ASD way. Overall, girls and women with ASD struggle socially in ways that differ from males; a strong drive to connect and be liked can make exchanges look more reciprocal, reducing concern, especially when academics are intact. This understanding helps explain why there are higher rates of missed or mistaken diagnoses among girls and women (Hiller et al., 2016; Sedgewick et al., 2019; Black et al., 2024; Paolizzi et al., 2025).

Cognitive profiles in ASD. It is also important to understand how differences in learned sociocommunicative skills play a critical role in shaping cognitive profiles, further emphasizing the need for not only therapy-based treatment differences but also school interventions (Paolizzi et al., 2025). Research shows that for both neurotypical and neurodivergent individuals, socio-communicative and social interaction skills contribute to language acquisition and the development of reasoning abilities, shaping cognitive profiles (Jarrold, 2003). Despite broad recognition of heterogeneity in autistic cognitive profiles, most research, much like work on social interactions, focuses primarily on boys with ASD. Verbal and nonverbal skills show discrepancies across autistic individuals, with visuospatial abilities often a relative strength and weaknesses commonly emerging in Processing Speed and Working Memory (Wilson, 2024). However, accounting for sex differences may yield different results. Given that social abilities influence cognitive profiles and that girls/women and boys/men with ASD often present differently socially, there is reason to expect sex differences in cognitive profiles. For example, despite a small sample size, a recent study of school-aged individuals with ASD found sex differences in cognitive profiles and cognitive flexibility, with girls showing better processing speed, whereas males performed better on visuospatial tasks, contrary to the common generalization of processing-speed weakness in ASD (Paolizzi et al., 2025).

Even though some studies have begun to show differences in cognitive profiles, most interventions for individuals diagnosed with ASD center around the assumption that visuospatial abilities are a relative strength; however, emerging sex differences blur this generalization (Ankenman et al., 2014). For instance, TEACCH is a common intervention that emphasizes using visual aids to help break down daily structure and tasks for those with ASD (TEACCH Autism Program, n.d.). However, if girls with ASD have faster processing speed, TEACCH’s instructional pace may feel slow, its heavy visual scaffolds overbearing, and its limited verbal back-and-forth understimulating, leading to disengagement or underestimation of their abilities. This is only one example of many intervention programs developed to address challenges generalized to males with ASD.

## 6. Misdiagnosis in Girls and Women with ASD

As stated above, current research highlights that many girls and women with ASD go undiagnosed. Even further, many of these same girls and women, particularly those who do not display language, obvious social, or intellectual impairments, are often misdiagnosed with other psychiatric or neurodevelopmental conditions. Misdiagnosis can lead to individuals receiving clinical interventions that do not address primary concerns, potentially resulting in ineffective treatment (Dell’Osso & Carpita, 2023; Lockwood Estrin et al., 2021).

Eating disorders (ED) are a common diagnosis girls receive instead of ASD, as symptomology in both diagnoses may overlap in rigid, ritualized behavior around food or intense special interests in diet or weight (Dell’Osso et al., 2023). However, if an individual is diagnosed with an ED rather than ASD, or not identified as having both, treatment that only targets the ED may risk not incorporating the clear structure, predictable routines, and slower pacing that are ideal for those with ASD (Li et al., 2022). Additionally, as mentioned before, girls and women with ASD use camouflaging in order to appear ‘neurotypical’. Even when camouflaging becomes apparent in girls, many are diagnosed as having Social Anxiety Disorder (SAD) rather than ASD (Dell’Osso et al., 2023). Further, core ASD traits such as inflexibility, rigid thinking, and heightened sensitivity to sensory stimuli may manifest as anxiety or panic in girls and women. This can mask not only underlying ASD features but can also lead to diagnoses of SAD and various other anxiety disorders, as these autism traits are often internalized rather than recognized as part of a neurodevelopmental profile (Dell’Osso et al., 2023).

Obsessive–compulsive disorder (OCD) is another common diagnosis that many individuals with ASD may be misdiagnosed with, as both can be characterized by the presence of repetitive behaviors (O’Loghlen et al., 2024). It is vital for clinicians to understand how repetitive behaviors may appear similar but function for different reasons in OCD versus ASD individuals. For instance, studies have seen that repetitive behaviors associated with OCD relate more to anxiety, as partaking in a repetitive or compulsive behavior reduces anxiety for an individual. However, for those with ASD, the relationship between anxiety and behavior is less clear, as studies show mixed results regarding whether repetitive behaviors cause or reduce anxiety in those with ASD (Jiujias et al., 2017). Further, there is a link between repetitive behaviors and challenges around inhibitory control in those with OCD, whereas those with ASD demonstrate repetitive behaviors more as a consequence of difficulties with set-shifting or switching their attention to another activity (Jiujias et al., 2017). Even though various studies have highlighted the differences in repetitive behaviors between these two diagnoses, many studies do not specifically look at differences in sex within those with OCD and ASD, and how that relates to repetitive behaviors. In a 2024 systematic review, no study looked at differences in sex or gender as a factor in repetitive behaviors between OCD and ASD, despite evidence that men with ASD, compared to girls and women with ASD, may show repetitive behaviors in diverse ways (O’Loghlen et al., 2024). This gap in the literature on sex differences may contribute to why OCD and ASD can look similar on standardized assessment, especially in terms of repetitive behaviors, which can lead to misdiagnosis.

Besides anxiety and obsessive–compulsive-related disorders, recent work has pointed to the potential for girls to be incorrectly misdiagnosed with Borderline Personality Disorder (BPD). The two diagnoses have similar symptomatology, including emotion regulation problems, identity/self-concept difficulties, and social reciprocity issues (Dell’Osso et al., 2023). In continuation, both BPD and ASD individuals, especially girls and women, show elevated risk to trauma, including bullying, interpersonal violence, and sexual abuse, which can exacerbate symptoms. Interestingly, when looking at research in BPD, autistic traits are overrepresented in BPD samples (Dell’Osso et al., 2023), yet since BPD is a “go-to” diagnosis for girls and women with chronic relational and emotion regulation problems, girls and women often get misdiagnosed with BPD rather than ASD. Understanding that ASD presents very differently, not only in girls versus boys, but also across individuals, it is essential to recognize ASD female phenotypes and their common misdiagnoses. This can help clinicians distinguish true ASD traits from symptoms of other conditions, ensuring that each person receives appropriate intervention and can reach their full potential.

## 7. A New Assessment Model: Multimodal Autism Assessment for Girls and Women (MAAGW)

A multimodal, expansive, inclusive approach. Perhaps one way to decrease both missed and misdiagnoses for girls and women is to consider more expansive and inclusive approaches to the overall assessment process. Given that girls, young women, and women tend to be less diagnosed, along with the reality that they may present very differently than what we typically diagnose as ASD, it is recommended that assessment prior to diagnosis takes a multi-modal approach that incorporates various data points and reports. Being diagnosed with ASD in a timely manner helps mitigate risks of emotional, behavioral, social, economic, and occupational difficulties (Wong et al., 2015). The literature suggests that a timely diagnosis may be less likely for girls and young women, given the way the two-factor model of ASD may be experienced by them (and observed by others). Accordingly, a more expansive and inclusive approach to assessment and diagnosis is suggested.

The following points contribute to a new assessment model to gender considerations in ASD assessment that Clauss-Ehlers introduces as the Multimodal Autism Assessment for Girls and Women (MAAGW). The MAAGW is a six-point model that encourages us to employ more expansive approaches to the assessment of ASD in girls and women that includes an emphasis on community diagnosis, self-report inclusion, testing at different developmental stages, tests that reflect the individual’s unique experiences, addressing the potential for camouflaging, and being aware of how RBBIs may show up for girls and women. Each is briefly described below.

Emphasis on community diagnosis. One area of exploration concerns the way in which a diagnosis of ASD is given to begin with. Currently, the “gold standard” for a confirmatory diagnosis of ASD is to administer one of the main testing measures and determine whether the individual meets the cutoff score for ASD (D’Mello et al., 2022). The main testing measures often include the Autism Diagnostic Interview Schedule, 2nd edition (ADOS; Lord et al., 2012) or the Autism Diagnostic Interview-Revised (ADI-R; Rutter et al., 2003). However, because the cutoff score in these measures ultimately determines whether an individual meets criteria for ASD, it can sometimes override the fact that the person already has a community diagnosis (D’Mello et al., 2022).

A community diagnosis refers to a diagnosis made by a practitioner based on ASD criteria described in the DSM-5-TR and informed by a range of data points, such as parent report, teacher report, self-report, medical records, and clinical interviews, among other sources of information (D’Mello et al., 2022). Interestingly, in their work on how gender is included in research, D’Mello et al. (2022) state that because females tend to score lower on the ADOS, which results in not meeting the cutoff score, they are not diagnosed with ASD. This results in many girls and women not being included in ASD research. In contrast, when a community diagnosis was incorporated, the outcomes of ASD assessments proved to be more equal in terms of men and women because the community diagnosis captured the ASD diagnosis. They state: “Reliance on community diagnosis rather than confirmatory diagnostic assessments resulted in significantly more equal sex ratios. These results provide evidence for a ‘leaky’ recruitment-to-research pipeline for females in autism research” (D’Mello et al., 2022).

While the work of D’Mello et al. (2022) is focused on the inclusion of girls and women in ASD research, it presents important clinical implications related to ASD assessment processes. Given the different understandings of what criteria means for girls and women, and how the “gold standard” might not capture the varied way this criteria is experienced by them, assessments that take a community diagnosis approach rather than a confirmatory diagnosis approach may likely better reflect ASD as it encompasses diverse data points from the girl’s or woman’s community, in addition to administering testing measures.

Self-report inclusion. As part of community diagnosis, self-report can be emphasized as an essential part of the assessment process. The age of the individual being assessed may determine the extent to which self-reports are included. For instance, for young children, the focus is often on interviewing the individual’s parent(s) and teacher(s) at school, as well as observing the child in the school setting. However, given the work of Henderson et al. (2023) that captures how DSM-5-TR (American Psychiatric Association, 2013) criteria might be reflected in the experiences of people and with ASD, it is suggested that self-reports may be a central aspect of the ASD evaluation. Further, it is suggested that self-report measures be used across the lifespan whenever developmentally appropriate, from childhood through adulthood. In this way, girls and women can share their experiences across different developmental stages. For less verbal examinees, drawing or illustrating feelings and responses may further elaborate the clinical picture (Kotroni et al., 2018; Mokhtari & Parchini, 2023; Round et al., 2017). A recommendation to include the voices of all examinees, across ages and genders, is suggested. Given unique challenges in receiving an accurate diagnosis, this seems particularly important.

Testing at differing developmental stages to address false negatives. A false negative is when someone is incorrectly told that they do not have a condition, when they do. For instance, a test says you do not have ASD when you do. As described below, ample literature demonstrates that girls and women may engage in camouflaging behaviors that lead to the absence of an ASD diagnosis (Hull et al., 2019) or a false negative. As shared above, social struggles related to ASD may only emerge when the social demands of an environment increase (e.g., navigating middle and high school) and the level of teacher and parental involvement typical of toddlerhood and early childhood decreases (e.g., classroom behavioral scaffolding, parent-supervised playdates), making it more difficult for individuals to engage in camouflaging. Hence, the developmental shifts in how ASD may present itself may coincide with developmental shifts in a young person’s social development. This suggests that if testing done at a younger age indicated a lack of ASD, then re-testing at different developmental stages such as adolescence and young adulthood is critical to address the potential for a false negative.

Incorporating tests that reflect the examinee’s unique presentation. From self-reports and reports from the community, it naturally follows that testing measures used to assess ASD for girls and women correspond with the unique experiences that are presented. For instance, an 18-year-old might share the following in her self-report: “I can use eye contact, but it never feels natural to me.” This may alert the examiner to the possibility of camouflaging. By incorporating tests that reflect what the examinee is telling us, the tester might decide to use the Hull et al. (2019) Camouflaging Autistic Traits Questionnaire (CATQ). Because girls and women might mask and compensate for social and communication difficulties, testing measures such as the ADOS-2 might not pick up on the less obvious ways that ASD is present. To help avoid a false negative with this type of presentation, assessment practices can be expanded to include observations of the examinee in day-to-day situations such as the school setting, incorporate more assessment tools into the overall assessment, and specifically ask about the impact of social interactions and how they are experienced.

Addressing camouflaging during assessment. Given that camouflaging can conceal ASD characteristics through the adoption of neurotypical behaviors, it is essential that camouflaging be evaluated alongside more standard assessment measures. For instance, the CAT-Q (Hull et al., 2019) is helpful to include in assessment batteries. While solely implementing the CAT-Q is not enough to conclude or exclude ASD, it may help clinicians make an accurate diagnosis (Hull et al., 2019).

Consideration of (RRBIs’ reevaluation as applied to gender. As mentioned, RRBIs are a core diagnostic area of ASD, defined as stereotyped or repetitive motor movements and inflexible interests, routines, or verbal and nonverbal patterns (Rubenstein et al., 2015; Fulceri et al., 2016; Duvekot et al., 2017; Lockwood Estrin et al., 2021; Rynkiewicz et al., 2016; Tillmann et al., 2018). Girls and women with ASD have been observed to have differing restricted interests from males, as well as decreased stereotypes across all age groups (Antezana et al., 2019). For instance, Antezana et al. (2019) conducted an experimental study using the Repetitive Behavior Scale-Revised (RBS-R; Lam & Aman, 2007), a self-report questionnaire that measures repetitive behavior in individuals with ASD. The items found to be in lower frequency in girls in comparison to boys were hand/finger fixations, object usage, preoccupations with parts of an object, and fascination or preoccupation with movement. Items in greater frequency in girls included hand/skin pulling, scratching/rubbing oneself, hoarding/saving, and insistence on sitting in the same spot. Some items reaching near significance of higher frequency in girls with ASD were washing/cleaning, self-care, disliking changes in others’ behavior and appearance, and strong attachment to an object (Antezana et al., 2019).

Across numerous studies on ASD, RRBIs are also reported to be less frequent and severe in girls and young women in comparison to boys and young men (Rubenstein et al., 2015; Lockwood Estrin et al., 2021). This means girls and young women with ASD have fewer stereotypical behaviors, routines, and rituals in comparison to males with ASD (Rubenstein et al., 2015; Lockwood Estrin et al., 2021). However, researchers also caution that this lower frequency of female RRBIs may be due to an unexplored understanding of how RRBIs manifest in the female phenotype of ASD (Rubenstein et al., 2015; Rynkiewicz et al., 2016; Tillmann et al., 2018).

Table 2 presents the MAAGW approach, which compares more traditional assessment techniques with newer ways that assessment might more accurately reflect the experiences of girls and women. The six-point model is presented that includes an approach to assessment for diagnosis, who is reporting on autism experience/observations, developmental considerations for when testing should occur, what assessments are to be included in the protocol, understanding observations within the potential context of camouflaging, and understanding how RRBIs might manifest for girls and women.

## 8. Gender Considerations Related to Accommodations for Individuals with ASD

School Accommodations. Research shows that girls and women with ASD are less likely to complete secondary school (Baumbusch et al., 2025; Stark et al., 2025). For example, a study that reported this trend examined a cohort of 3802 students (652 girls) with ASD within a school system in British Columbia, Canada. The authors examined longitudinal trends in students with ASD from kindergarten through grade 12 with regard to graduate rates, as well as receiving three additional high school credentials (e.g., BC Adult Graduation Diploma, BC Certificate of Graduation, and BC School Completion Certificate, combined) (Baumbusch et al., 2025). The authors found that girls with ASD had lower graduation rates and hypothesized that the results may be due to male-concentrated data (e.g., more males were represented) (Baumbusch et al., 2025). These results further suggest that camouflaging may make it difficult to identify girls with ASD because of their disproportionately low representation. Another study by Stark et al. (2025) longitudinally observed the entire student cohort in Stockholm County, Sweden (N = 736,180), and similarly found lower secondary school completion rates. Girls with ASD had lower completion rates of upper secondary school than neurotypical females, and this rate was much higher than the rates of boys with ASD in comparison to neurotypical boys (Stark et al., 2025). Other studies found that girls are placed at lower rates than boys in specialized schools that meet the needs of ASD (van den Helder et al., 2025). Building further on this trend, a study utilizing 138 individuals across all levels of education found that teachers reported concerns of ASD at significantly lower rates in girls (Hiller et al., 2014). Narrowing the focus to primary education, Whitlock et al. (2020) found that girls with ASD reach out for general assistance at a lower frequency compared to boys with ASD. The paper addressed reaching out as a form of support-seeking, including contacting the school, an educational psychologist, and a medical professional.

Due to the findings from these studies, gender-aware proactive support is needed at various levels, along with a defined need for greater recognition of ASD in schools. To ensure schools are held accountable for supporting girls with ASD in the classroom, one prospective option is a mandatory reporting and training system that may lead to improved outcomes and help address gender disparity in ASD diagnoses (Maciver et al., 2025). Accompanying this new training and reporting system is an implementation system that tracks data down to classrooms, ensuring educators continue to operationalize their training at the classroom level. School districts should record ASD outcomes and identification rates of girls on a timely basis, while also exploring cross-sectionality between socioeconomic correlations, individualized education programs, and other potential factors due to discovered socioeconomic trends between ASD and educational placement (Gindi, 2019), as well as other unprecedented factors. Our reasoning behind exploring additional trends is to potentially reveal new dimensions within the data. Some hypothetical examples include difficulty for girls with ASD in commuting or particular distractions from learning in certain classrooms. This knowledge generation will allow further accommodations to be made.

Accommodations to address camouflaging. As mentioned, camouflaging presents itself in high rates of girls and women with ASD due to expectations set in neurotypical societal environments. In one study, 12 of 14 women were viewed as not having ASD by professionals, and 8 of 14 women reported “camouflaging” or “pretending to be normal” (Bargiela et al., 2016). Establishing flexibility in uniform, sensory accommodations, quiet or noise-reduced lunches, and alternatives to participation may decrease rates of camouflaging and potentially improve identification of ASD signs. This is supported by multiple studies that provide evidence that visual, auditory, and other sensory factors in classrooms have an impact on the education of students with ASD (Petersson-Bloom & Holmqvist, 2022). It is essential that professionals are aware that camouflaging is more common and prominent in girls and women with ASD, and that accommodations should reduce or eliminate pressure to act neurotypically (Milner et al., 2023; Cancino-Barros et al., 2025). Teachers, psychologists, and other professionals in school settings must not overlook that girls with ASD in early adolescence have higher rates of vocal expressiveness (Corbett et al., 2021), potentially leading to assumptions that these more social girls are not interested in environments with less socialization.

A potential solution is to reduce the social risks of decreasing masking and camouflaging by making social and sensory recovery options available for interested students, especially females. Studies back this claim through findings that demonstrate camouflaging in general education classrooms is increased around unfamiliar peers and teachers, carrying subsequent emotional and attentional costs, potentially even taking away from learning (Halsall et al., 2021; Goscicki et al., 2025). Examples of successful social and sensory recovery options include a quiet area or a mindfulness retreat without expected conversation, eye contact, or work (MacLennan et al., 2023). For children with ASD, sensory rooms with auditory, physical, and visual stimulation may provide a benefit (Unwin et al., 2022).

Mentoring as an accommodation. Young people with ASD may feel isolated from their peers in academic environments due to perceived differences from neurotypical peers (Hebron et al., 2015; Urbaniak & D’Amico, 2025). As a potential solution, schools can dedicate resources to establishing peer groups and mentoring by students, teachers, trained staff, or even other girls and classmates with ASD. Other neurodivergent individuals may share insights into their experiences, and through this, girls with ASD may learn more about identity, disclosure, and social expectations within the school setting (Urbaniak & D’Amico, 2025).

Accommodations in the workplace. Misconceptions of women with ASD in the workplace are often common (Quintero et al., 2025). For instance, in a cross-sectional survey of 880 employees examining attitudes towards coworkers with ASD, respondents misinterpreted RRBIs as a symptom of ADHD rather than ASD, and, notably, 60.6% believed that workplaces were not adequately suited for individuals with ASD (Quintero et al., 2025). Given these gaps in knowledge and awareness, Quintero et al. (2025) argue that increased targeted education is needed. This aligns with our view that interventions tailored to how ASD may present in girls and women are warranted to build workplace awareness and inclusion.

In another study that included 55 adults with ASD, 32 of whom were women, results indicated that social expectations varied by gender in the workplace (Hayward et al., 2022). For instance, findings suggested that women with ASD had different social demands and greater camouflaging pressure, potentially contributing to greater levels of stress. The authors recommend that employers consider how environments may create strain with the possibility of incorporating supports that decrease masking and promote inclusive environments (Hayward et al., 2022).

Further, Hayward et al. (2018) reviewed studies published over 13 years with participants who were high-functioning women with ASD. Findings indicated high levels of unemployment among women who participated in the studies. Additionally, they reported that many of the women with ASD felt they were not adequately challenged despite a strong willingness to work. The review concluded that this may have been due to reported communication difficulties, challenges in social workplace relationships, and sensory issues, suggesting gender-based structural changes are important ways to decrease pressure for masking, overstimulation, and improvements in the job-search process (Hayward et al., 2018). In alignment with exploring potential accommodations, one study interviewed employees with ASD from Poland, approximately 31% of whom were women, to explore the advantages of remote work for people with ASD (Tomczak et al., 2022). These authors found that research participants reported fewer instances of sensory overload, fewer social demands, and flexibility suited to their needs (Tomczak et al., 2022). Research on girls’ and women’s experiences in the workplace suggests structural changes are needed to decrease misconceptions related to ASD and to establish supports for sensory, social, and camouflaging demands.

Challenges related to higher education. A potential challenge for girls and women who have been diagnosed with ASD, as well as for those who are not yet diagnosed, concerns the transition to college life. For many youths in the United States, college is a time of greater independence, with students engaging in a range of courses, studying with different professors, and deciding upon or testing out a major. For students living on campus, other adjustments include negotiating having roommates or living in a single room, building a campus community, and developing a peer network. Given these developmental changes, it is important to note that at the very time that young adults are faced with building social relationships in a new environment and often without the presence of adult scaffolding, is also often the time that young adults do not have the services and supports they might have had for ASD in high school if, for instance, the student had an Individualized Education Plan (IEP) that hopefully transferred into accommodations at the college level.

The contrasting experience between a greater need for social skills vs. lesser ASD intervention and support on college campuses is an area of accommodation that warrants systemic response. In October 2024, Jane Theirfeld Brown, director of College Autism Spectrum, shared the following with U.S. News & World Report: “In 2000, there were two specialized autism support programs, and now in 2024, there are close to 100…Colleges are really listening and looking at enhanced services and programs” (Nesbit, 2024). The College Autism Spectrum website has a section where 2-year and 4-year postsecondary programs throughout the 50 states and Washington, D.C. can post campus programs that offer support for students with ASD. As of November 2025, 91 colleges and universities had posted their information on the College Autism Spectrum website. While programs are increasingly being implemented on campus, some programs require an additional fee for students to participate. More programs are needed across campuses to meet the needs of ASD students with affordable options for participation. Additionally, training teachers, administrators, and staff in how ASD might manifest for young adult women is a campus-wide approach to building awareness and outreach.

## 9. Limitations of the Narrative Review

This narrative review has limitations specifically related to gender. The focus of the current narrative review is on gender-related considerations for girls and women. A limitation is that this focus does not consider transgender and non-binary people. It is important to highlight the ways in which a male-centered history has shaped ASD and the lack of responsiveness to the experiences of girls and women. At the same time, it is important for the field to explore experiences of ASD among transgender and non-binary people. In regard to the screening process of including/excluding literature for review, potential biases were reduced by involving all three authors. However, it is possible that subjective interpretations of the inclusion criteria may have inadvertently influenced the final selection.

## 10. A Call for a Female-Focused Neurodiversity Paradigm

Given the varied ways that ASD is experienced by girls and women, we suggest building on Walker’s (2012) neurodiversity paradigm to explore how it plays out among this group. A female-focused neurodiversity paradigm employs us to look at strengths and individual differences rather than take a disease-laden approach, to truly listen to how girls and women describe their experiences, and to reframe assessment, intervention, accommodations, and understanding as building on strengths rather than looking at ASD as a deficit. A Female-Focused Neurodiversity Paradigm includes the following:

### 10.1. Research Agenda

#### 10.1.1. Directions for Future Research

As shared above, more research is needed that provides data about the experiences of women and girls with ASD. Future research can operationalize and explore the nature of RRBIs for women and girls, the role of camouflaging and masking, developmental considerations, and interventions that would provide support, among other research foci. Research is also needed about the experiences of transgender and non-binary people who have been diagnosed with ASD.

#### 10.1.2. Inclusion of Girls and Women as Research Participants

Research findings indicate that factors such as cutoff scores exclude girls and women from research studies. As a result, much of our current data on ASD is based on the experiences of boys and men. To generate knowledge about the experience of ASD among girls and women, they need to be included in research studies.

#### 10.1.3. Funding for ASD Research Focused on Girls and Women

Proposed directions for future research, including the inclusion of girls and women in research studies, can be furthered through a funding agenda. Dedicated funding for research on the experience of ASD among girls and women can support the output of new data in this domain. This is also important as empirical data has implications for clinical practice with practitioners providing interventions that correspond to an evidence base.

#### 10.1.4. Dissemination of Research Knowledge

The research generated on the experience of ASD among girls and women can be disseminated to constituency groups such as practitioners, educators, parents, families, caregivers, communities, students, universities, other researchers, and policymakers, among other groups. In this way, a unified system can provide services, educational environments, parenting/caregiver practices, accommodations, research, and policies that reflect the evidence presented as it relates to ASD among girls and women.

### 10.2. Policy Agenda

#### 10.2.1. Policy Development and Implementation Responsive to the Experience of ASD Among Girls and Women

Research knowledge and awareness can shape policy development and implementation in communities and organizations. Policies can correspond with the experiences of ASD among girls and women, such as providing workplace accommodations and educational settings that support neurodiversity and a sense of belonging. Community groups within schools, universities, and social networks can implement policies that foster a sense of inclusion.

#### 10.2.2. Inclusion of Girls and Women with ASD in Policy Development

Just as ASD research needs to include girls and women, so too do policies need to reflect their voices. Given the differences and varying nuances of experience described above, it is important to include girls and women with ASD as those who can speak to policy changes that would be helpful for them.

### 10.3. Clinical Agenda

#### 10.3.1. Clinical Training

Clinical training is often lacking in its focus on ASD in general, leaving recent graduates of clinical training programs with little experience or knowledge about neurodiversity. Training programs can begin to offer courses focused on neurodiversity, including learning about the experience of ASD among girls and women.

#### 10.3.2. Workshop Experiences in Educational Environments

Given the prevalence of ASD, along with the realities of underdiagnosis and misdiagnosis, schools and universities can provide in-house service training focused on ASD in general, including an emphasis on ASD among girls and women. Because educators such as classroom teachers and professors are in contact with students every day, such in-service training can further their understanding of what they may observe in the classroom, written assignments, and social interactions. Through this awareness, educators can engage in dialogue with students and families about potential outreach or referral.

#### 10.3.3. Clinical Practice

It is hoped that building a greater evidence base of research and supporting clinical development with training focused on ASD, clinical practice will incorporate interventions that are in alignment with the needs and experiences of girls and women with ASD. Providing clinical interventions like counseling and psychotherapy where the client feels heard and understood provides an important resource and support. Additionally, if a client with ASD is struggling with navigating social situations such as transitions to middle school, high school, and/or college, the attuned therapist can help tease out which aspects might relate to social and communication challenges related to ASD and which struggles might reflect the transition period, or a combination of both. In this way, therapy can help girls and women navigate social concerns that accompany critical life transitions.

#### 10.3.4. Testing and Assessment

The testing and assessment agenda supports an expansive process as outlined in the MAAGW model. A community diagnosis, incorporating multiple data points and testing and assessment, draws on a range of perspectives that inform understanding. Including measures that specifically examine camouflaging and masking address how ASD may show up for girls and women.

Taken together, a female-focused neurodiversity paradigm that includes research, policy, and clinical agendas seeks to augment our understanding of the experience of ASD among girls and women. It is from this understanding that we can better identify, address, intervene, and most of all, support girls and women with ASD.

## 11. Conclusions

The current article explores how ASD research has historically focused on individuals assigned male at birth, leading to diagnostic tools normed on male samples. As a result, these tools often fail to capture how ASD manifests in girls and women, contributing to misdiagnoses, exclusion from research, and a lower likelihood of meeting DSM-5-TR criteria for ASD. Building on this, the article further reviews research showing that girls and women with ASD tend to present with subtler and more heavily masked or camouflaged traits, particularly in social interactions, which often leads to them being overlooked during assessment. Additionally, emerging work on cognitive functioning suggests distinct cognitive profiles between the sexes, including evidence that some girls and women with ASD may show stronger processing speed than boys and men with ASD, challenging the male-based assumption that visuospatial strengths and processing-speed weaknesses may characterize ASD broadly (Wilson, 2024). Differences also emerge in the RRBI domain, where the behaviors of girls and women may be more internalized or more socially normative, which can potentially lead clinicians to miss symptoms that do not resemble stereotypically “male” presentations (Rubenstein et al., 2015; Tillmann et al., 2018). Together, these differences in ASD presentation have resulted not only in underdiagnosis but also in frequent misdiagnosis of girls and women with ASD. Both underdiagnosis and misdiagnosis lead to a lack of intervention and treatment responsive to ASD.

The recognition that girls and women are often mis- or underdiagnosed, alongside a clearer understanding of the female autism phenotype, calls for a better conceptualization of how ASD presents for them across the DSM-5-TR two-factor model (e.g., nonverbal communication, social reciprocity, sensory issues, and RRBIs). Addressing these limitations requires clinicians to reinterpret diagnostic criteria through a female lens. This understanding of differences in presentation across sexes highlights the need for assessment, diagnostic, and accommodation practices to be modified to accurately reflect both male and female ASD phenotypes. The Multimodal Autism Assessment for Girls and Women (MAAGW; Clauss-Ehlers, 2026) was introduced to guide a more inclusive and accurate assessment of ASD in girls and women. This model recommends a multimodal, community-based assessment approach that incorporates self-report, developmental trajectory, contextual observations, and camouflaging measures (e.g., the CAT-Q). The MAAGW can help guide clinicians toward a more valid, gender-aware diagnostic decision, which hopefully will directly reduce both missed and incorrect diagnoses. To further close this gap, it is necessary to provide accommodations that meet the needs of girls and women. Accommodations must reflect sensory, social, and communication needs while reducing masking pressure (e.g., quiet spaces, sensory-friendly environments, non-social breaks). A neurodiversity paradigm can then help clinicians, researchers, and providers think about ASD in terms of how symptomology reflects individual differences among girls and women with a focus on difference as difference, not pathology (Parritz & Troy, 2024).

Further, after examining current research, it is clear that expanding studies to include a sufficient number of both sexes is vital for helping clinicians and researchers understand the diversity of autistic presentation through the lens of sex differences. As such, investigating female-specific RRBIs and how they differ in presentation can help clinicians identify these patterns in girls and women rather than attributing them to another diagnosis. In line with this, exploring how camouflaging interacts with the presentation of cognitive profiles and social symptoms can help tailor assessments to account for these variations in presentation. Expanding this research can help develop assessment instruments that are normed for female autism phenotypes and better account for intersectionality (e.g., SES, race/ethnicity, gender identity) in girls and women with ASD identification and outcomes. We hope that understanding sex differences within the scope of ASD can not only provide more tailored interventions but can also help all individuals with ASD reach their full potential.

## Figures and Tables

**Table 1:** Autism Criteria Summary Chart (table by Donna Henderson, PsyD; 2026, reprinted/shared with Dr. Henderson’s permission).

**Social/Communication (Need All Three):**			
**Criterion**	**What People Think of:**	**But it Really Includes Differences with:**	**Those with a Less Obvious Presentation MAY:**
1. Differences in reciprocating social or emotional interactions.	Complete lack of reciprocity and empathy. Disengaged.	Responding to name. Offering greetings, responding to greetings. Initiating conversation. Conversing about a variety of topics. Taking turns (not monologuing or interrupting). Sharing info, feelings, and objects. Responding to praise. Picking up “social breadcrumbs,” i.e., responding to subtle social bids. Having a typical social filter. Intuitively understanding others’ thoughts, feelings, intentions (particularly regarding non-autistic people). Managing conversations that involve more than two people. Amount of energy required to prepare for and navigate social interactions. Difficulty recognizing subtext in conversation. Using context to manage interactions, e.g., how much or which detail to include, what social norms apply.	Show more typical social behavior, particularly in one-on-one structured situations such as evaluations or therapy. Be able to camouflage skillfully; camouflaging may hide social differences. Be more engaged and chattier. Be more reciprocal or have an easier time being reciprocal when talking about topics of interest. Follow scripted rules for politeness. Have an inner experience of effort, confusion, or other experiences during social interactions. Experience a high need for recovery time after interactions.
2. Nonverbal communication differences.	No eye contact. Flat affect.	Using eye contact to manage interactions (more than just meeting someone’s eyes). Volume, intonation, prosody. Body posture. Personal space. Flat or unusual affect. Nonverbals may not vary or may be exaggerated, not integrated, or may not be typical for context. May show differences interpreting nonverbals, particularly of non-autistic people.	Have fairly or even completely typical expressive nonverbal presentation (e.g., typical eye contact, body language, voice intonation, etc.) Have a negative experience of eye contact and other nonverbals, e.g., effortful, distracting, unpleasant.
3. Differences in developing or maintaining relationships.	Doesn’t have friends. Doesn’t want friends.	Reciprocal play. Understanding relationships. Making and keeping friends. Deepening and sustaining friendships. Engaging in relationships at typical developmental level (i.e., best friend, groups, flirting, etc.) Social motivation. Social flexibility. Conflict management.	Differences in play may be more subtle. Engage in more pretend play but may be scripted or have more focus on setting up scenes. Timeline! Social differences may not be apparent until social demands exceed abilities. May have close friends, especially when they share interests. Patterns in relationships, e.g., always connecting with younger or older kids or adults, quirky people, highly flexible people.
**Repetitive/Restricted (Need two of four):**			
**Criterion**	**What people think of:**	**But it really includes:**	**Those with a less obvious presentation MAY:**
1. Repetitive or unusual speech, movements, or use of objects.	Flapping or echolalia	Wide variety of motor movements (not just flapping). Lining up or organizing toys or other objects. Idiosyncratic phrases. Scripted or formal language, e.g., mother instead of mom. Pronoun reversal. Unusual noises or humming. Toe walking (after about age four). Watching the same movie/TV show, listening to the same music, reading the same book many times (not just 2 or 3 times). Repetitive picking, hair pulling or twirling.	Have fewer and more subtle repetitive behaviors. Common examples include pacing, twirling (body or hair), and repetitive use of objects, but there are endless possibilities. Tend to systematize, such as making lists, spreadsheets, or other ways of systematizing information.
2. Flexibility.	Routines or rituals. Behavioral problems.	May experience differences in flexibility, e.g.,: Difficulty with transitions. Difficulty with change. Strong preference for sameness. Obsessive mind, perfectionism. Black-and-white thinking. Unusually strong moral compass. Unusually strong rule following. Difficulty adjusting when reality does not fit one’s expectations of a situation. Difficulty using context to aide flexibility. On/off switch; tendency to shut down. Uneven ability to understand humor; literal. Difficulty parting with objects that may seem useless to others, e.g., candy wrappers.	Differences in flexibility may be internalized not necessarily externalized. Experience anxiety related to change. Be strongly focused on details. May not intuitively adjust by using context.
3. Intense or unusual interests.	Overtly odd interests (e.g., memorizing airport codes).	Interests that are atypical AND/OR intense. Drive for exhaustive exploration; desire/need for significant depth of information in areas of interest. Can also have unusually strong attachment to specific objects.	Fewer and less obvious atypical interests than those with more easily identified autism. Tend toward typical interests but at an intense level. Common examples are animals, social justice, pop stars, anime, K-pop, fan fiction, and makeup. In adults, autism itself can be an intense interest. The intensity may manifest internally rather than overtly talking about the interest. Interests can be used as social or occupational currency. Interests may be about people or animals rather than information or objects.
4. Sensory differences.	Atypical sensory craving (e.g., looking at fans); sensitivity to loud noises.	Sensory hyper-responsivity, even if only when young. May present as enhanced perception. Sensory hypo-responsivity, such as unusually high tolerance for pain. Includes all eight sensory systems; consider proprioception and vestibular differences. Particularly important to consider differences in interoception. May explain hygiene challenges, clothing choices, unusual phobias, and/or limited diet.	Sensory differences may not be apparent to others. Interoception may be atypical, which can manifest as hypo- and/or hyper-responsivity to homeostatic feelings, such as hunger and thirst, which can affect self-care and self- regulation. Differences in interoception may also affect one’s ability to identify their affective emotions, which can result in alexithymia.
**Additionally, two more criteria must be considered:** First, **symptoms must be traced back to childhood**. The DSM-5 states that “symptoms must be present in the early developmental period.” And, the ICD-11 states that “the onset of the disorder occurs during the developmental period, typically in early childhood.” However, they both also state that symptoms may not fully manifest until later, “when social demands exceed limited capacities.” The DSM-5 also adds that symptoms may not be seen at times because they “may be masked by learned strategies.” This all highlights the fact that autism is not something that can begin in adolescence or adulthood. It is important to determine whether the issues were present but manageable early in life. Be careful to avoid assuming that a symptom wasn’t present when the person may have been hiding it from others (masking/camouflaging) and/or if the parents were highly accommodating. Second, a diagnosis of autism spectrum disorder requires evidence of **clinically significant impairment** in social, occupational, or other important areas of current functioning. This can include being exhausted when forced to mask or camouflage, with a significant need for recovery time alone. It is common for the presentation to differ depending on context, holding it together when in public and breaking down once at home. Other areas that may be impacted include anxiety, depression, burnout, school refusal, difficulty launching into adulthood, activities of daily living, etc. Clearly, individuals who are seeking either an evaluation or therapy do have clinically significant challenges.			

**Table 2:** A new assessment model: Multimodal autism assessment for girls and women (developed by Clauss-Ehlers, 2026; original table by Caroline S. Clauss-Ehlers, PhD, ABPP).

Traditional Approach	Multimodal Approach
Confirmatory Diagnosis	Community Diagnosis, in addition to Confirmatory Diagnostic Measures
Parent Report; Teacher Report; some Self-Report	Self-Report Central to Assessment, in addition to Collateral Reports
Testing at One Developmental Point to Confirm or Rule Out Diagnosis	Testing at Varying Developmental Stages when Environmental Social Demands may Exceed the Individual’s Social Resources
Standard Protocol	Testing Protocol that Reflects the Unique Experiences of the Examinee; Observation of Examinee in Social Situations (e.g., school setting); Incorporate More Assessment Tools; Ask Questions Specific to How Social Interactions are Experienced
Taking Observations at Face Value	Address and Assess for Potential Camouflaging Behaviors
Focus on Well-Known Restrictive Repetitive Behaviors and Interests (RRBIs)	Engage in Observation and Inquiry about Subtle RRBIs (girls having interests that teachers may describe as “‘seemingly random’ (e.g., rocks, pens stickers)” (Harrop et al., 2015).

## Data Availability

No new data were created or analyzed in this study.

## Appendix A. Summary of Autism Spectrum Disorder DSM-5-TR Criteria with Examples That Might Reflect the Experiences of Females (Original Table Created by Caroline S. Clauss-Ehlers, PhD, ABPP Based on Sources Presented Below)

Table based on sources from:

- American Psychiatric Association (2022). Diagnostic and Statistical Manual of Mental Disorders (5th ed., text rev.). Author. https://doi.org/10.1176/appi.books.9780890425787.
- Centers for Disease Control and Prevention (2025, May 8). Clinical Testing and Diagnosis for Autism Spectrum Disorder. https://www.cdc.gov/autism/hcp/diagnosis/index.html (accessed on 16 January 2026).

**A**	**B**	**C**	**D**	**E**	**Severity**
A. “Persistent deficits in social communication and social interaction across multiple contexts” (American Psychiatric Association, 2013, p. 50) in the following three areas:	B. “Restricted, repetitive patterns of behavior, interests, or activities” (American Psychiatric Association, 2013, p. 50) in at least two of the following areas:	C. “Symptoms must be present in the early developmental period (but may not become fully manifest until social demands exceed limited capacities, or may be masked by learned strategies later in life)” (American Psychiatric Association, 2013, p. 50)	D. “Symptoms cause clinically significant impairment in social, occupational, or other important areas of current functioning.” (American Psychiatric Association, 2013, p. 50)	E. “These disturbances are not better explained by intellectual disability (intellectual developmental disorder) or global developmental delay. Intellectual disability and autism spectrum disorder frequently co-occur; to make comorbid diagnoses of autism spectrum disorder and intellectual disability, social communication should be below that expected for general developmental level” (American Psychiatric Association, 2013, p. 51)	Severity
1. “Deficits in social-emotional reciprocity” (American Psychiatric Association, 2013, p. 50); e.g., difficulty engaging and sharing back and forth in ongoing conversation; isolating in conversation rather than engaging and sharing interests, etc.	1.“Stereotyped or repetitive motor movements, use of objects, or speech” (American Psychiatric Association, 2013, p. 50); e.g., engaging in the same activity over and over again, like watching the same TV show or reading the same book				**Level 1:** “Requiring support” (American Psychiatric Association, 2013, p. 52)
2. “Deficits in nonverbal communicative behaviors used for social interaction” (American Psychiatric Association, 2013, p. 50); e.g., may engage in camouflaging behaviors so people are not aware of social deficits; may engage in eye contact but may feel strained in doing so	2. “Insistence on sameness, inflexible adherence to routines, or ritualized patterns of verbal or nonverbal behavior” (American Psychiatric Association, 2013, p. 50); e.g., becoming very upset at even small changes in routine or expectations, feeling anxious about changes to the daily routine				**Level 2:** “Requiring substantial support” (American Psychiatric Association, 2013, p. 52)
3. “Deficits in developing, maintaining, and understanding relationships” (American Psychiatric Association, 2013, p. 50); e.g., may struggle with making and maintaining consistent friendships over time; may insist on being friends with someone without having known the person for a long time; may not be aware about how to build trust for maintaining friendships	3. “Highly restricted, fixated interests that are abnormal in intensity or focus” (American Psychiatric Association, 2013, p. 50); e.g., having an overly intense level of interest in something like baking, make-up, or some other area				**Level 3:** “Requiring very substantial support” (American Psychiatric Association, 2013, p. 52)
	4. “Hyper- or hyporeactivity to sensory input or unusual interest in sensory aspects of the environment” (American Psychiatric Association, 2013, p. 50); e.g., hyper-reactive to noises, responding as though they are very loud when to others they might not be, hyporeactive to pain, not responding to a pain stimulus as one might be expected to				
